# Advances in Zika Virus–Host Cell Interaction: Current Knowledge and Future Perspectives

**DOI:** 10.3390/ijms20051101

**Published:** 2019-03-04

**Authors:** Jae Kyung Lee, Ok Sarah Shin

**Affiliations:** Department of Biomedical Sciences, College of Medicine, Korea University Guro Hospital, Seoul 08308, Korea; jae.lee0321@gmail.com

**Keywords:** Zika virus, cellular targets, innate immune evasion, cell death

## Abstract

Emerging mosquito-transmitted RNA viruses, such as Zika virus (ZIKV) and Chikungunya represent human pathogens of an immense global health problem. In particular, ZIKV has emerged explosively since 2007 to cause a series of epidemics in the South Pacific and most recently in the Americas. Although typical ZIKV infections are asymptomatic, ZIKV infection during pregnancy is increasingly associated with microcephaly and other fetal developmental abnormalities. In the last few years, genomic and molecular investigations have established a remarkable progress on the pathogenic mechanisms of ZIKV infection using in vitro and in vivo models. Here, we highlight recent advances in ZIKV-host cell interaction studies, including cellular targets of ZIKV, ZIKV-mediated cell death mechanisms, host cell restriction factors that limit ZIKV replication, and immune evasion mechanisms utilized by ZIKV. Understanding of the mechanisms of ZIKV–host interaction at the cellular level will contribute crucial insights into the development of ZIKV therapeutics and vaccines.

## 1. Introduction

Infectious diseases have gained importance as a significant threat to public health following the recent outbreaks of arthropod-transmitted viruses (arboviruses) in the western hemisphere. Global emergence of arboviruses such as dengue virus (DENV), chikungunya, yellow fever virus (YFV), and Zika virus (ZIKV) has become possible due to several factors including urbanization, rapid population growth, and climate change [1]. Although environmental changes have given importance to the movement of the human population, especially by air travel, the extent to which humans have reshaped the environment has also led to a dynamic spread of pathogens and their vectors. The dissemination of arboviruses, in particular, is dependent on their vectors, and reports of these pathogens in new global destinations should raise concerns of the expansive distribution of the vectors, such as the *Aedes* species of mosquitoes. Of the numerous human pathogens that have experienced a rapid geographic distribution, this review focuses on ZIKV, which has raised international health concerns due to its broad spectrum of transmission routes, autoimmune disorders in adults, and neurodevelopmental complications in newborns [2,3]. ZIKV belongs to the *Flaviviridae* family along with Japanese encephalitis virus (JEV), West Nile virus (WNV), and DENV, all of which are medically important viruses transmitted by mosquitoes or ticks. As of January 2018, PAHO has reported 223,477 confirmed ZIKV cases that have cumulated worldwide between 2015 and 2018 [4]. Despite the global distribution of ZIKV, there are no clinically approved vaccines or therapeutic treatments available to combat the infections [5]. As a result, international concern surrounding ZIKV in terms of control, treatment, and prevention has classified the virus as a global threat to public health.

Although most cases of ZIKV infection result in asymptomatic or mild flu-like symptoms, such as fever, rash, and conjunctivitis, the series of outbreaks that started in Yap islands in 2007 has emphasized just how wide of a phenotypic spectrum of disease can be caused in humans by the virus. Recent incidences of infection have resulted in severe phenotypes including Guillain–Barré syndrome, meningoencephalitis, and fetal abnormalities such as microcephaly and spontaneous abortion [6]. Up to date, diagnosis of ZIKV infection has depended on molecular and serological testing, employing ELISA and RT-PCR platforms for IgM and RNA detection accordingly [7]. However, these methods of diagnosis are only useful for virus detection within a short frame from the symptom onset as levels of viral RNA and IgM antibodies decline over time. Laboratory testing of infants suspected of congenital ZIKV infection includes detection of viral RNA in serum and urine, and IgM antibodies in serum and CSF of infants [8]. Although tests should be performed as early as possible once an infection is suspected, the optimal timing and type of specimen and assay for detection remain undetermined. 

Since the series of outbreaks in 2007, ZIKV infection has increasingly been associated with cases of neurological complications and congenital malformations. Microcephaly, which is defined by a brain size that is at least two standard deviations below the mean, is one clinical presentation of a congenital ZIKV infection, and the World Health Organization has already established an etiological link between ZIKV infection and birth defects like microcephaly in Brazil [7,9]. Congenital abnormalities induced by ZIKV infection have confirmed the possibility of vertical transmission. Despite the prevalence of birth defects, diagnosis of congenital microcephaly remains a challenge due to the existence of various etiological factors involved [10]. Pathogenesis studies have confirmed the neurotropism of ZIKV, although the exact molecular mechanism of neuropathogenesis remains unclear [7]. In addition to microcephaly, exposure to ZIKV during pregnancy can result in visual and hearing impairments in the newborn [11]. Thus, the phenotypic spectrum of outcomes of pregnancy-associated ZIKV infection has identified ZIKV as a dangerous and atypical member among the flaviviruses. 

Flaviviruses carry out their life cycle by utilizing machinery and functions of the host cell [12]. Consequently, flavivirus–host cell interactions are essential for the pathogenesis. Many of these crucial interactions, however, remain elusive. In this Review, we focus on ZIKV, a member of the *Flaviviridae* family, and highlight the viral pathogenesis at the level of cellular mechanisms and interactions.

## 2. Structure of ZIKV

**Figure 1:**
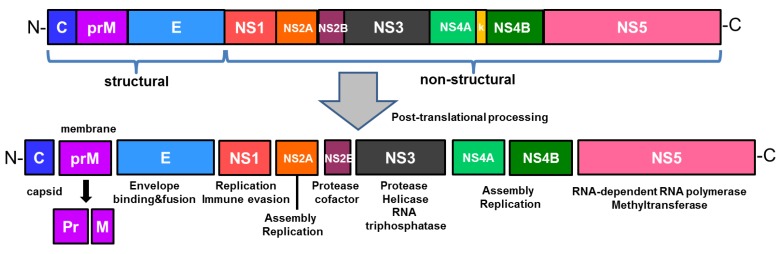
The structure of Zika virus (ZIKV) genome and its encoded proteins. The single open reading frame encodes a polyprotein precursor that is post-translationally cleaved into three structural proteins (capsid, membrane and envelope) and seven nonstructural proteins (NS1, NS2A, NS2B, NS3, NS4A, NS4B, and NS5).

ZIKV is a single stranded, positive sense RNA virus whose genome encodes a single polyprotein that is cleaved into three structural proteins, such as capsid (C), membrane precursor (prM), envelope (E) proteins, and seven nonstructural (NS) proteins (NS1, NS2A, NS2B, NS3, NS4A, NS4B, NS5), as shown in Figure 1 [5]. While the virus is comprised of structural proteins, nonstructural proteins are responsible for viral replication and assembly [5,13]. The structure of ZIKV is similar to that of other flaviviruses. Flaviviral NS1 is involved in viral replication and infection, and interacts with the host immune factors when secreted extracellularly for immune evasion and pathogenesis [14,15,16]. In addition, NS1 has been suggested as a biomarker in diagnosis of flaviviruses such as DENV, which results in early stages of infection corresponding to high levels of NS1 secretion [17]. NS3 consists of a protease domain, which is linked to NS2B to form a protease complex in the case of ZIKV, and a helicase domain [18]. As the largest and most conserved protein of the flavivirus genus, NS5 plays an important role in viral replication. ZIKV NS5 contains an RNA dependent RNA polymerase (RdRp) domain, which is essential for viral replication, and a methyltransferase (MTase) domain, which is involved in translation and evasion of host immune response [16]. The envelope (E) protein, which mediates viral entry, of ZIKV possesses a highly conserved fusion loop region that is also found in WNV, YFV, and DENV1-4 [13,19]. As a result, the fusion loop serves as a target for antibodies generated against broad range of flaviviruses like DENV and ZIKV [13,20]. Furthermore, transposon mutagenesis screening has demonstrated the capacity of ZIKV to tolerate genetic diversity, as evident from ZIKV E gene’s tolerance of mutations, and this genetic flexibility within a viral genome has important implications on evasion mechanisms of adaptive immunity [21]. 

Flaviviruses have the unique ability to produce subgenomic flavivirus RNA (sfRNA) from the 3’ untranslated region (UTR) of the genomic RNA, and studies have shown that sfRNA not only is required for efficient virus replication, but also contributes to cytopathic effect (CPE) and pathogenicity [22,23]. Although it is unclear why flaviviruses, unlike other members of the *Flaviviridae* family, evolved a mechanism to generate sfRNA, this noncoding RNA may be involved in regulating host antiviral response through RNA-mediated pathways. Pijlman et al. has shown that a reduction in CPE of viruses results from the failure to produce sfRNA1, a finding that suggests the role of sfRNA1 in inducing cell death following a virus infection [23]. Although exact mechanisms and pathways of cell death involving sfRNA require further research, sfRNA is another potential viral factor utilized by flaviviruses to promote host cell death.

## 3. Cellular Entry and Targets of ZIKV

Since 2016, scientific communities have dedicated rapid and extensive research efforts on ZIKV in terms of its molecular pathogenesis and associated cellular signaling pathways and factors. Table 1 shows a comprehensive list of currently identified susceptible human cells to ZIKV infection. ZIKV exhibits broad tropism, and the localization of its cellular targets that have been identified so far range from the brain, placenta, and skin, to testis, kidney, and retina. The entry of ZIKV is facilitated by the following phosphotidylserine receptors, such as TIM (TIM1, TIM4) and TAM (Axl, Tyro3) in human primary trophoblasts [24]. Because mosquito bite remains the major transmission route, cells localized in the epidermis and dermis were primarily considered as targets for ZIKV infection [25,26]. 

Upon entry of the host cell by endocytosis, ZIKV releases a viral genome that undergoes replication and virion assembly for further infection [12]. ZIKV infection exhibits broad distribution and persistence in body tissues and fluids, a characteristic that distinguishes ZIKV from other arthropod-transmitted flaviviruses. For example, the presence of ZIKV has been detected in the amniotic fluid of pregnant women and in semen, suggesting the possibility of sexual and perinatal transmissions, both of which are routes that other arboviruses have not exhibited [27]. Furthermore, pathogens detected in the amniotic fluid are most likely to cause infections during pregnancy and pose a threat to both the mother and fetus [27]. As previously mentioned, microcephaly is a severe consequence of ZIKV infection in the prenatal brain. Despite the existence of various etiological agents of microcephaly, cell death of premature differentiation-induced depletion of neural stem cells (NSCs) is one possibility [28]. Tang et al. [29] was able to confirm that neural stem and progenitor cells were preferentially targeted by ZIKV. Furthermore, the capability of ZIKV to infect human neural progenitor cells (NPCs) has been confirmed in several studies. ZIKV exhibits preferential infection of neural progenitor cells, leading to cell-cycle arrest, increased apoptosis, and inhibition of NPC differentiation. All of these outcomes following ZIKV infection are associated with delayed development and birth defects, such as microcephaly in the mouse model [30].

Prenatal infection of ZIKV has adverse pregnancy effects that range from congenital abnormalities to pregnancy loss and miscarriage [48,49,50]. Although the placenta possesses innate immunity to defend against infiltrating pathogens, ZIKV infection is able to overcome this defense and cross the maternal–fetal barrier. The placenta provides a passage for viruses to carry out vertical transmission, during which placental macrophages called Hofbauer cells may take part in spreading ZIKV in the placenta and promoting vertical transmission of ZIKV [36]. In accordance, El Costa et al. identified the first trimester placenta as a permissive target of ZIKV, as evident from confocal microscopy analyses of first trimester trophoblasts, cytotrophoblasts, syncytiotrophoblasts, and extravillous trophoblasts, and Hofbauer cells, suggesting that these cells provide a pathway for ZIKV to reach fetal cells, resulting in a congenital infection [51]. Although studies have established the vital roles of these immune cells during pregnancy, especially in context of congenital ZIKV infection, further research is needed to determine the exact mechanism that ZIKV employs to infect the placental immune cells and its contribution to miscarriage and fetal dysfunctions.

Sexual transmission poses a concerning threat to people who are outside of the ZIKV epidemic-prone regions. Previous knowledge of flaviviruses as exclusively vector-borne diseases was contested when a case of ZIKV infection resulted in a human-to-human transmission in 2008, between a husband and his wife [52]. Since then, increasing reports of sexual transmission of ZIKV, most of which are characterized by male to female transmission, have been documented [53]. Molecular and serological testing of samples from infected patients revealed infectious viral load of ZIKV in semen [51]. Furthermore, the prolonged presence of ZIKV RNA differentiates ZIKV from other flaviviruses because the immune response usually clears the viral nucleic acid within a short timeframe after symptom onset. Mansuy et al. describes cases of ZIKV infection in which viral RNA was detected up to 141 days after the onset of symptoms, far surpassing the 37 day limit for positive detection in urine and blood samples [51]. Sexual transmission of ZIKV also results in a longer incubation period compared to acquisition of the virus through a mosquito bite, which results in a mean incubation period of 6 to 15 days [54,55,56]. Such discrepancies in the incubation period may result from differences in the timing and/or site of ZIKV infection, both factors that can affect the tropism and propagation mode exhibited by the virus.

Immunohistochemistry has also detected ZIKV in the head of spermatozoa, which does not express the previously described entry receptor Axl [57]. Therefore, Sertoli cells, which mediate the differentiation of germ cells into spermatozoa and display high expression level of Axl, are hypothesized to be involved in transmitting ZIKV to spermatozoa [57]. In Yockey et al. (2016), the vagina was identified as another site susceptible to ZIKV infection, and sustained a viral replication that was more robust than other organs [58]. Although rare, sexual transmission of ZIKV cannot be overlooked as this route has implications on ZIKV infection in relation to male fertility, pregnancy, and viral persistence.

ZIKV infection is also capable of breaching the blood–retinal barrier, as seen from the ocular symptoms. Although an immune privilege organ, an ocular ZIKV infection manifests as conjunctivitis and pan-uveitis, the former being the more common form of disease, in addition to induction of inflammation resulting from a pathogen-associated molecular pattern-activated response or the production of cytokines, cell death, and/or recruitment of leukocytes [59]. In mice, viral RNA was detected in tears, as well as multiple cell types of the eye including the retina, cornea, and iris, along with the optic nerve [60]. Ocular manifestations of ZIKV infection demonstrate viral pathogenesis in previously uninvestigated sites. Despite the tropism displayed by ZIKV infection for specific regions of the eye, further studies are necessary to outline the exact mechanisms of ZIKV-induced ocular disease. Evidence of ZIKV infection persisting in organs such as the eyes and testis in both human and animal models demonstrates that organs previously considered immune privileged are sites of viral replication and persistence [59,61,62]. 

Preferential targets of ZIKV infection exhibited high expression levels of entry receptor genes, such as Axl and heat shock protein genes, which were enriched in not only radial glia cells, but also microglia, astrocytes, and endothelial cells [28]. The phosphatidylserine receptor Axl on the cell surface mediates virus entry and the viral E proteins facilitate attachment to host cell [63]. Axl is a member of the Tyro3AxlMer (TAM) family of tyrosine kinase receptors whose functions consist of clearing apoptotic cells and regulating innate immunity [64]. Gas6 is a ligand of Axl that recognizes the viral particle and functions as a bridge between the virus and host cell [41]. The strong enrichment of Axl in radial glia of the human fetal cerebral cortex provides a possible explanation for why ZIKV selectively targets these neural stem cells and a potential mechanism for ZIKV-induced microcephaly. For example, Lemke et al. has identified two inhibitors of Axl, MYD1 and R248, that inhibit the ligand-receptor interaction and kinase activity of Axl, accordingly, to ultimately inhibit ZIKV infection [41]. It is interesting to note the dual role of Axl, which normally promotes NSC survival, replication, and neurogenesis, in addition to maintaining the blood-brain barrier and protecting against neurotropism [65,66]. Consequently, inhibiting Axl function to protect against ZIKV infection may have adverse outcomes that require consideration when identifying potential therapeutic targets. 

## 4. Multiple Cell Death Pathways Induced by ZIKV Infection

In addition to antiviral responses, a virus infection mediates changes in various cellular pathways, including cell survival, death and metabolism. Cell death pathways can be manipulated to benefit either the host by eliminating the pathogen, or the infecting pathogen by eliminating host immune cells [67]. Multiple cell death pathways, such as apoptosis, necroptosis, and pyroptosis, are utilized by the host and virus, and clearance or persistence of the virus depends on who manipulates the pathway. Crosstalk between different cell death pathways also provides protection for innate immune signaling pathways that are frequently targets of pathogen attacks. 

Various studies have confirmed the occurrence of neuronal apoptosis following a ZIKV infection in vivo and in vitro [68,69]. NSCs and NPCs, which display high susceptibility to ZIKV, undergo apoptosis pathways. Presence of fragmented nuclei and formation of autophagocytic vacuoles were visible beginning at 24hpi. In efforts to determine the reasons for the cell deaths, ZIKV structural envelope (E) protein, and its truncated forms, were suggested as a possibility of inducers [70]. Following the overexpression of the E proteins, inhibition of proliferation, cell cycle arrest, and apoptosis were observed. Among the several mechanisms of cell death, upregulation of p53, p21, along with cleavage of caspase 9 and caspase 3, demonstrated that apoptosis was the most likely mechanism of cell death in NSCs and NPCs. Wu et al. also describes the potential inhibitory role of ZIKV NS2B-NS3 (NS2B3) on apoptosis [71]. Overexpression of NS2B3 in HT1080 cells inhibited cleavage of caspase 3 and poly ADP ribose polymerase (PARP), and conferred resistance to poly (I:C)-induced cell death.

In Azevedo et al., apoptosis and necrosis were identified as the main pathways of neuronal cell death, resulting from the combined actions of immune factors and the virus [72]. Recent studies have confirmed the role of inflammasome during ZIKV-induced host immune response [73]. Immunohistochemistry analysis of brain tissues in Azevedo et al. showed significantly higher expression of NLRP1, NLRP3, and AIM2, cytokines IL-1β, IL-18, IL-33, and caspase 1 in cases of ZIKV-induced microcephaly, highlighting IL-33 as one of the cytokines that exerts multiple actions in relation to necroptosis, pyroptosis, and activation of inflammasome [72]. Furthermore, Monel et al. suggests paraptosis as another form of cell death induced by ZIKV infection, during which formation of large cytoplasmic vacuoles, which were derived from the endometrium reticulum, were visible in various cell types, including Hela cells, primary human astrocytes, and skin fibroblasts [74]. As mentioned above, multiple cellular death pathways have been suggested to be induced upon ZIKV infection. It will be interesting to further study an array of virulence factors to modulate host cell death pathways as a component of the survival strategy. 

## 5. Host Cell Restriction Factors that Limit ZIKV Replication

Through active interference with viral replication in various ways, interferons (IFNs) orchestrate the primary response to viral infection [75]. Their origin and cellular targets distinguish the three types of IFNs (I, II, III), all of which engage with their respective receptors to initiate signaling through the JAK/STAT pathway, and induce activation of interferon-stimulated genes (ISGs) [76]. Upon activation, ISGs directly target factors of the virus life cycle to control the infection [77]. All three types of IFNs are implicated in mediating the antiviral state of the host immune response. 

Belonging to the same flavivirus genus, DENV and ZIKV share similarities beyond phylogeny, including host immune responses. Proteins of the type I IFN subgroup (including IFN- α, β, ε, τ, and δ) exhibited contradictory roles in DENV pathogenesis, during which certain proteins were upregulated compared to other that were reduced in infected patients [78,79]. For type II IFNs, elevated serum levels of dengue patients were associated with higher chances of experiencing increased severity of disease [79,80,81,82,83]. In vitro studies of type III IFNs induction following DENV infection demonstrated that IFN-λ has the inhibitory potential to control DENV replication [84]. In Bayer et al., IFN- λ1 of the type III IFN family (λ1-4) conferred protection against ZIKV infection in placental trophoblasts, mediating antiviral signaling at the interface between the mother and fetus [37]. Syncytiotrophoblasts display constitutive expression of IFN- λ1, which acts in both autocrine and paracrine manner to protect other syncytiotrophoblasts and non-placental cells from ZIKV infection [37]. According to Chaudhary et al., ZIKV infection had differential effects on type I and type II IFN responses, as demonstrated by the ability of ZIKV NS5 to suppress type I and type III IFN while activating type II IFN signaling in a luciferase reporter assay [85]. The differential regulation of type I and type II IFNs in turn result in selective expression of ISGs and differentiated activation of immune and proinflammatory responses [85,86,87]. 

Taking advantage of the fact the absence of type I IFN signaling leads to successful viral pathogenesis, viral replication can be inhibited by inducing ISGs as in the case of small membrane-associated interferon-inducible transmembrane proteins (IFITMs) [88]. IFITMs have demonstrated the ability to inhibit viral replication of various pathogens including flaviviruses such as WNV and DENV [88,89,90,91,92]. In Savidis et al., gain- and loss-of function studies have revealed IFITM1 and IFITM3 as inhibitors of ZIKV, and IFITM3 specifically inhibited the early stages of ZIKV replication, evident from the significant decrease in the expression level of viral RNA. Accordingly, IFITM3 may be responsible for mounting an initial defense against ZIKV infection even before the induction of interferon signaling and downstream ISGs. As seen from the studies involving these proteins, IFITMs provide a possible mechanism for the prevention and treatment of viral infections including ZIKV [88,89,90,91,93]. 

Small non-coding RNAs are another potential restriction factor that can limit ZIKV replication. MicroRNA (miRNA) have been shown to have a role during ZIKV infection by regulating a broad spectrum of cellular processes [94]. Because a single miRNA has multiple targets within a specific cellular process, a change in expression level induced by a virus infection results in an amplified effect [95,96]. In Smith et al., high content immunofluorescence screening identified miRNA34 to inhibit the replication of three flaviviruses (DENV, WNV, and JEV) through the Wnt/β-catenin signaling pathway, during which a dampened Wnt signaling leads to TBK1-induced phosphorylation of IRF3 and initiation of downstream IFN signaling [94]. It will be interesting to further study the antiviral role of differentially regulated small RNAs during ZIKV infection.

## 6. Immune Evasion Mechanisms Utilized by ZIKV

**Figure 2:**
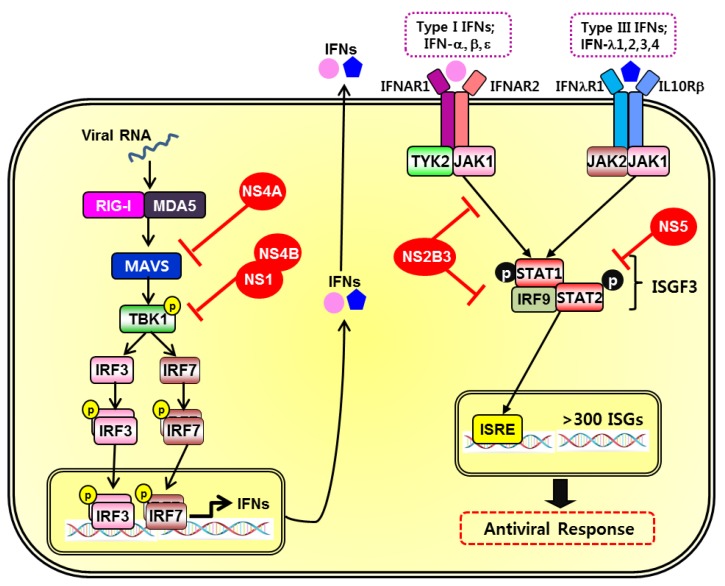
Innate immune evasion mechanisms by ZIKV. During ZIKV infection, viral RNA sensors and interferon-mediated signaling through downstream adaptor molecules and transcription factors can be targeted for immune evasion strategies. Viral proteins, indicated by red color, can interfere with interferon (IFN) responses by suppressing the induction of signaling pathways at multiple steps [26,71,98,99,100,101,102].

The efficient replication of flaviviruses is associated with the ability of the virus to inhibit the induction of IFNs and downstream signaling pathways. Consequently, viruses employ multiple immune evasion mechanisms to mount an effective countermeasure to the host cell immune response. Nonstructural proteins, for example, interact with molecules involved in innate immunity to establish efficient viral replication. ZIKV infection leads to the inhibition of type I IFN production and downstream interferon stimulated genes (ISGs) [97]. ZIKV NS1, NS4A, and NS4B proteins function as the main suppressors of type I IFN induction through the suppression of TBK1/IRF3 pathways. Figure 2 describes diverse mechanisms by which ZIKV evade innate immune responses. 

Nonstructural proteins have roles during the virus life cycle and directly interact with signaling proteins of the innate immune response. Sironi et al. has emphasized the selective pressures faced by NS1 in flaviviruses and its important functions in viral RNA replication and immune evasion, as seen during the pathogenesis of DENV [98]. The amino acid differences in NS1 proteins may explain the difference between the virus-host interaction of flaviviruses, and how the host immune response is differentially modulated to viruses such as ZIKV in comparison to DENV. Furthermore, by interacting with host immune components like the RIG-I-like receptors, NS1 proteins have demonstrated a crucial function in flavivirus pathogenesis [26,98]. NS4A suppresses type I IFN induction via inhibition of MAVS interaction as a way of immune evasion [99]. NS4B contains a N-terminal region that blocks IFN response through suppression of IFN-α and β signaling [100]. 

Furthermore, NS1 and NS4B of ZIKV inhibit production of type I IFN by targeting TBK1, preventing its oligomerization, and in turn stabilize NS2B-NS3 [71]. Consequently, NS2B3 inhibits the downstream signaling of type I IFN, JAK-STAT, by promoting JAK1 degradation, in addition to inhibiting ZIKV-induced apoptosis, as previously mentioned, in a manner independent of NS2B3-mediated Jak1 degradation. The focus on WNV NS5 has been centered on its ability to inhibit JAK/STAT pathways and this context allows for a better understanding of the mechanism for immune evasion [101,102]. Similarly, NS5 of ZIKV directly interacts with and induces degradation of STAT2, which is a transcriptional activator acting downstream of type I and type III IFNs that has been targeted by various viruses closely related to DENV [97,103]. In Kumar et al., immunoblot and confocal microscopy analysis confirmed that unlike the levels of STAT1 that remained unchanged, the levels of STAT2 were nearly abrogated following ZIKV infection [97]. While ZIKV NS5 is utilized for evasion of type I IFN response, this protein also selectively activates type II IFN, IFN-γ, signaling to induce inflammation [85]. NS5 also initiates and facilitates flavivirus replication by forming a replication complex and interacting with other nonstructural proteins [104]. 

Among the cellular pathways implicated in development of the brain and regulation of autophagy, Akt-mTOR pathway is hijacked by pathogens to benefit viral replication in host cells. Akt-mTOR pathway has also been identified as a target of ZIKV proteins NS4A and NS4B for neurogenesis inhibition and autophagy induction [105]. DENV and ZIKV both induce autophagy through NS4 protein in order to suppress Akt-mTOR signaling in the host cell. The suppression of Akt-mTOR signaling pathway leads to an upregulation of autophagy and impaired neurogenesis in the host cell, ultimately resulting in enhanced viral replication. Taken together, the nonstructural proteins of ZIKV work synergistically to restrict host antiviral response at multiple levels and interfere with important cellular survival and homeostasis mechanisms for to evade the host immune system.

## 7. Vaccines and Therapeutics

The reemergence of ZIKV has posed a serious threat to global public health and has prompted urgency for the development of effective vaccines and therapeutic treatments against ZIKV infection. Animal models have demonstrated the efficacy of various vaccine platforms, several of which have advanced to human trials [106,107]. Furthermore, modified mRNA encoding ZIKV prM-E and live-attenuated ZIKV with NS1 mutations studied in pregnant mice showed promising potential to protect against placental and fetal ZIKV infection, preventing congenital ZIKV syndrome [107]. Richner et al. demonstrated that vaccination with the two previously described platforms protected against challenge with ZIKV during pregnancy, as evident from the following: reduced level of viral mRNA in maternal, fetal, and placental tissue, and absence of virological evidence of transmission in majority of cases [107]. Based on the findings of current vaccine platforms that have sufficient immunity against ZIKV infection in animal models, a more comprehensive evaluation of the candidates warrant an effective vaccine against the virus in the future.

In addition to the lack of preventable methods against ZIKV infection, the increased pathogenicity of the virus has triggered an extensive investigation of candidates for anti-ZIKV drugs and therapies. Currently, design and development of drugs targeting either the virus or factors of the host immune response are in progress, and these inhibitors range in target from viral capsid, nonstructural proteins, including NS5, specifically RdRp and methyltransferase domain inhibition, NS3 helicase, and NS2B-NS3 protease, to nucleotide analog inhibitors, nucleoside biosynthesis inhibitors, in addition to the previously mentioned IFITMs [108]. Viral entry and replication have shown to be inhibited by the following drugs and drug-like molecules: Obatoclax, squalamine, cavinafungin, nanchangmycin, dyramycin-biotin, ZINC33686641, and ZINC49605556 [108]. Sofosbuvir (Sovaldi), which terminates synthesis of viral RNA prematurely, is one example of a drug that has been undergone clinical phase I and phase II trials, during which it has proven its safety and efficacy [108,109]. Drug repurposing, a process during which a library of drugs that have already been FDA-approved is screened for potential ZIKV inhibitors, has also risen as a viable alternative that saves time that would have been spent in design, development, and clinical testing of novel drugs. Chloroquine, quinacrine, mefloquine, and GSK369796 are some of the drugs that have shown promising potential through various screening methods [110,111,112]. Furthermore, the ability of ZIKV to cross barriers into sites previously considered as immune privileged is another concern that addresses the urgency for pregnancy-safe drugs to become available. 

The use of natural compounds has also been an alternative antiviral approach. For example, polyphenols, such as delphinidin and epigallocatechin gallate have exhibited potential antiviral activities against flaviviruses including ZIKV, DENV, and WNV [113]. A member of the largest family of polyphenols, the flavonoid isoquercitrin also demonstrated antiviral effect against ZIKV by acting on viral entry [114]. A bacterially derived nanchangmycin is another natural compound that inhibited ZIKV entry in human osteosarcoma cells (U2OS), human brain microvascular endothelial cells, and human trophoblast cell line Jeg-3, and was also active against DENV, WNV, and chikungunya virus [115]. Furthermore, berberine and emodin showed strong inhibitory effect on ZIKV infectivity in vitro [116].

## 8. Future Directions for ZIKV Studies

Although remarkable progress in ZIKV research has created opportunities for the development of vaccines and therapeutics, some of which are currently advancing through clinical trials, the following key outstanding questions still remain to be addressed and should be the focus of future perspectives: 1) what is the mechanism by which ZIKV crosses the placenta and infects developing fetus? 2) What host and viral factors contribute to ZIKV persistence in the placenta and other immune-privileged sites? 3) What are the roles of noncoding RNAs during ZIKV infection? 

Although animal models provide useful platforms for supplementing and elevating cell-based in vitro studies, these techniques fail to reflect the physiological parameters and cellular communication occurring in human conditions, which are essential to understand the mechanisms of development and disease. The lack of human-derived models has given rise to organoids and organ chips that rely on a combination of microfluidic and microfabrication techniques. Organoid technology and organ-specific chip models, such as placenta-on-a-chip and eye-on-a-chip, are able to mimic the interactive contributions made by major cell types involved in virus pathogenesis and overcoming the limitations of in vitro models [51,117,118,119,120]. Further insights into virus–host cell interactions and mechanisms associated with increased virulence of ZIKV will better aid in the design and development of safer and more potent drugs and therapeutics against ZIKV. 

## Figures and Tables

**Table 1:** Cellular targets and potential entry receptors for ZIKV.

Origin	Cell Targets	Potential Entry Receptor	References
Skin	Epidermal keratinocytes	Axl, Tim-1, Tyro3	[25,26]
Skin	Dermal fibroblasts	Axl, Tim-1, Tyro3	[25,26]
Blood	Dendritic cells	DC-SIGN	[31]
Blood	Monocytes	Unknown	[32,33]
Placenta	Hofbauer cells	Axl, Tyro3, TIM1	[34,35,36]
Placenta	Trophoblasts	Axl, Tyro3, TIM1	[24,37,38]
Placenta	Endothelial cells	Axl, Tyro3, TIM1	[39]
Brain	Neuronal progenitor cells (NPCs)	Axl, TLR3	[28,40]
Brain	Astrocytes and glial cells	Axl	[41,42]
Retina	Retinal pericytes	Axl, Tyro3	[43]
Retina	Retinal microvascular endothelial cells	Axl, Tyro3	[43]
Testis	Spermatozoa	Tyro3	[44,45]
Testis	Sertoli cells	Axl	[44,46,47]
